# 

**Published:** 1890-11

**Authors:** 


					﻿NOVEMB-RR, 1
OLD SERIES
VoU XXV
e£Jk®us$*
? (855 4
NEW SERiiiS
* Vol. VI I.-No. J.
TTMT
EM®
IM
^H. V. M. MILLER, M.D., LL.D.
VIRGIL 0. HARDON, M.D.
F. W. McRAE, M.D., M’n’g’ng Ed.
JAMES P.HARRISOIS
t. Atlanta,ga.
SUBSCRIPTIONS $2.60 A YEAR.
				

